# A comparison of peripapillary vessel density between subjects with normal-tension glaucoma and primary open-angle glaucoma with similar extents of glaucomatous damage

**DOI:** 10.1038/s41598-023-36369-w

**Published:** 2023-06-07

**Authors:** Min-Woo Lee, Hwa-Young Yu, Kee-Sup Park, Sun-Young Jin, Jin-Soo Kim

**Affiliations:** 1 Department of Ophthalmology, Konyang University College of Medicine, Daejeon, Republic of Korea; 2 Modoo’s Eye Clinic, #238, Daedeok-daero, Seo-gu, Daejeon, Republic of Korea; 3 Department of Ophthalmology, Chungnam National University Sejong Hospital, #20 Bodeum 7-ro, Sejong, Republic of Korea

**Keywords:** Eye diseases, Ocular hypertension, Optic nerve diseases

## Abstract

The purpose of this study was to identify differences in retinal microvasculature impairments between patients with normal-tension glaucoma (NTG) and those with primary open-angle glaucoma (POAG) with similar extents of structural and visual field damage. Participants with glaucoma-suspect (GS), NTG, POAG, and normal controls were consecutively enrolled. Peripapillary vessel density (VD) and perfusion density (PD) were compared among the groups. Linear regression analyses were performed to identify the relationship between VD, PD and visual field parameters. The VDs of the full areas were 18.3 ± 0.7, 17.3 ± 1.7, 16.5 ± 1.7, and 15.8 ± 2.3 mm^−1^ in the control, GS, NTG, and POAG groups, respectively (P < 0.001). The VDs of the outer and inner areas and the PDs of all areas also differed significantly among the groups (all P < 0.001). In the NTG group, the VDs of the full, outer, and inner areas were significantly associated with all visual field parameters including the mean deviation (MD), pattern standard deviation (PSD), and visual field index (VFI). In the POAG group, the VDs of the full and inner areas were significantly associated with PSD and VFI but not with MD. In conclusion, with similar degrees of retinal nerve fiber layer thinning and visual field damage in both groups, the POAG group showed a lower peripapillary VD and PD than the NTG group. VD and PD were significantly associated with visual field loss.

## Introduction

Glaucoma is the leading cause of irreversible visual loss characterized by progressive optic neuropathy via degeneration of retinal ganglion cells, with an estimated more than 70 million people affected^1^. The optic nerve damage of glaucoma cannot be explained by only mechanical damage caused by high intraocular pressure (IOP)^2^. Previous studies reported that glaucoma progression could be related to the optic nerve hypoperfusion attributable to vascular dysfunction in patients with both high and normal IOPs^3–5^. Harris et al.^4^ reported that reductions in ocular blood flow were associated with morphometric changes in the optic nerve head, loss of visual function, and accelerated glaucomatous disease progression. Tobe et al.^5^ also found that a reduction in retinal blood flow (revealed using confocal scanning laser Doppler) was associated with structural glaucomatous progression. Therefore, studying changes in the retinal microvasculature and its circulation in patients with glaucoma is essential.

Optical coherence tomography angiography (OCTA) enables visualization of the fine microvasculatures of multiple retinal layers, and many OCTA studies have reported impairment of the retinal microvasculature in patients with glaucoma. Hou et al.^6^ reported that OCTA-based superficial macular vessel density (VD) was significantly lower in primary open-angle glaucoma (POAG) eyes than in healthy ones. Scripsema et al.^7^ found that the OCTA-measured annular perfused capillary density in normal-tension glaucoma (NTG) patients was significantly lower than that in normal controls. Although microvasculature impairment is evident in both NTG and POAG, the extents may differ because the pathological mechanisms of the two diseases may be different. Several studies have reported impairment of the retinal microvasculature in both eyes with NTG and POAG; however, studies comparing detailed microvasculature pathologies are lacking.

Here, we explored the differences in retinal microvasculature impairment betwee patients with NTG and those with POAG. We compared the peripapillary VD and perfusion density (PD) of eyes with NTG and POAG with similar extent of structural and visual function damage, as well as normal controls and glaucoma suspects (GS).

## Methods

### Patients

This prospective cross-sectional study adhered to the tenets of the Declaration of Helsinki and was approved by the Institutional Review Board/Ethics Committee of Konyang University College of Medicine, Daejeon, Republic of Korea. Patients who visited the glaucoma clinic of Konyang University Hospital were enrolled and examined from April 2021 to May 2022. Informed consent was obtained from all patients.

All subjects underwent detailed history-taking and a complete ophthalmic examination, including measurement of best-corrected visual acuity (BCVA) and IOP (via Goldmann applanation tonometry), gonioscopy, measurements of the spherical equivalent, axial length, the Humphrey visual field (HVF), spectral-domain optical coherence tomography (SD-OCT), and OCTA. Patients with GS, NTG, POAG, and normal controls (all over the age of 18 years) were consecutively recruited. All glaucoma diagnoses were confirmed by glaucoma specialists (S.Y.J. and K.S.P.) based on evidence of neural rim loss on dilated stereoscopic examination of the optic nerve, glaucomatous visual field defects apparent on standard automated perimetry (a cluster of ≥ 3 points with *P* < 0.05 on a pattern deviation map in at least one hemifield, including ≥ 1 point with *P* < 0.01; a pattern standard deviation of *P* < 0.05; or a glaucoma hemifield test result outside the normal limits, as confirmed by at least two examinations), and/or peripapillary retinal nerve fiber layer (pRNFL) loss evident on the peripapillary assessment of SD-OCT. Only reliable visual field tests (fixation loss ≤ 20%, false-positive ≤ 15%, false-negative ≤ 15%, no other evidence of poor quality, and no abnormal results caused by factors other than glaucoma) were included. The inclusion criteria for POAG patients were a history of pre-treatment elevated IOP (> 21 mmHg) and open angles on indentation gonioscopy. NTG patients were similarly required to exhibit open angles but to have pretreatment IOPs ≤ 21 mmHg. GSs were defined as having optic disc changes suspicious for glaucoma or an IOP > 21 mmHg without repeatable glaucomatous visual field changes^8^. A suspicious optic disc was defined as a disc with observable excavation, neuroretinal rim thinning or notching, or a localized or diffuse RNFL defect suggestive of glaucoma on optic disc stereophotographs^9,10^. The exclusion criteria were a history of any retinal or choroidal disease, secondary glaucoma, a history of ocular trauma, any prior intraocular surgery other than cataract extraction, any ocular disease including corneal disease, any retinal abnormality, any neuro-ophthalmic disease, a BCVA poorer than 20/40, and an axial length ≥ 26.0 mm. If both eyes in a participant met the inclusion criteria, one eye was randomly selected to avoid statistical bias.

### OCT and OCTA analysis

SD-OCT examination was performed using Cirrus HD-OCT (Carl Zeiss Meditec, Dublin, CA, USA). The RNFL thickness was measured using a 200 × 200 optic disc cube scanning protocol. The optic nerve head was placed at the center of the image during scanning, and a 200 × 200-pixel resolution axial scan was obtained over an area of 6 × 6 mm, including the optic nerve head and its surroundings. The RNFL thicknesses were calculated for the four quadrants (superior, temporal, inferior, and nasal) within the 3.46 mm-diameter scan circle. Images with a signal strength of < 7 and those exhibiting obvious decentration or segmentation errors were excluded.

**Figure 1:**
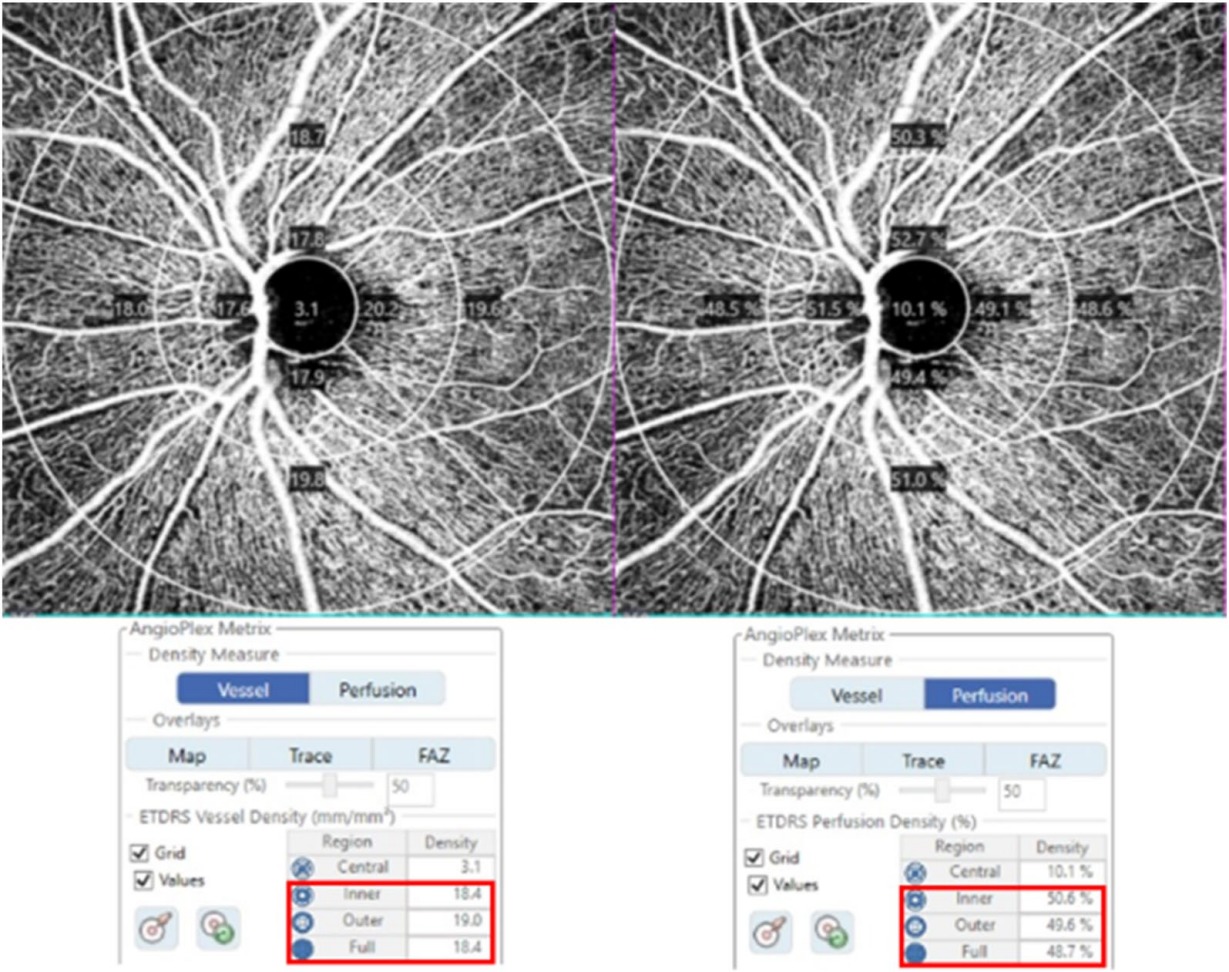
Optical coherence tomography angiography image (6 × 6 mm) centered on the optic disc. En face image of the superficial capillary plexus overlaid with the Early Treatment of Diabetic Retinopathy Study grid. Red boxes indicate the automatic measurements for vessel density and perfusion density of the inner, outer, and full areas.

OCTA was performed simultaneously with SD-OCT using a Cirrus HD-OCT 6000 device running the AngioPlex software (Carl Zeiss Meditec), with a wavelength of 840 nm taking 68,000 A-scan/s. Sensitivity and accuracy were assured using an optical microangiography (OMAG) algorithm and retinal tracking technology. We measured an optic disc-centered scan using a 6 × 6 mm pattern mode, which contains an approximate area of 6 × 6 mm over the optic nerve head. All scans were evaluated using the *en face* OCTA images automatically generated by the OMAG algorithm of AngioPlex software. The VD (total length of perfused vasculature per unit area in the region of measurement) and PD (total area of perfused vasculature per unit) of the superficial capillary plexus (which runs from the internal limiting membrane to the inner plexiform layer) were automatically measured. The software quantified VD and PD using the Early Treatment of Diabetic Retinopathy Study subfield. We evaluated peripapillary VD and PD in the superficial capillary plexus of both the inner and outer rings and in the full area (Fig. 1). Images exhibiting fixation loss, segmentation errors, motion artifacts, or signal strengths < 9 were excluded.

### Statistical analysis

Baseline demographics and OCT and OCTA measurements were compared using a one-way analysis of variance followed by the post-hoc Bonferroni test. The χ^2^ test was used to compare categorical data. Linear regression analysis was performed after adjusting for age, sex, BCVA, and axial length in the NTG and POAG groups to define the relationship between OCTA and visual field parameters. All statistical analyses were performed using SPSS software (version 22.0; IBM Corp., Armonk, NY, USA).

## Results

### Demographics

A total of 273 eyes were included in the study; 88 in the control group, 50 in the GS group, 73 in the NTG group, and 62 in the POAG group. Age, sex, spherical equivalent, and axial length did not differ significantly among the groups (Table 1). The BCVAs were − 0.02 ± 0.05, 0.06 ± 0.09, 0.06 ± 0.15, and 0.07 ± 0.13 (P < 0.001) (post-hoc: control < GS/NTG/POAG), and the IOPs were 14.9 ± 2.6, 15.0 ± 2.8, 12.4 ± 3.0, and 14.6 ± 3.3 mmHg (P < 0.001) (post-hoc: NTG < control/GS/POAG) in the four groups, respectively. In terms of visual field parameters, the average mean deviations (MDs) were − 1.74 ± 2.49, − 6.75 ± 7.80, and − 7.70 ± 7.93 dB, and the median MDs were − 1.17 (interquartile range [IQR], − 2.50 to − 0.22), − 4.33 (IQR, − 10.38 to − 1.65), and − 6.13 dB (IQR, − 13.17 to − 1.81) in the GS, NTG, and POAG groups, respectively (P = 0.005). On post-hoc analysis, the MD of the NTG group did not differ significantly from that of the POAG group (GS vs. NTG, P = 0.016; GS vs. POAG, P = 0.004; NTG vs. POAG, P = 0.737). The pattern standard deviation (PSD) and visual field index (VFI) results were similar (PSD: GS vs. NTG, P < 0.001; GS vs. POAG, P < 0.001; NTG vs. POAG, P = 0.527) (VFI: GS vs. NTG, P = 0.006; GS vs. POAG, P = 0.003; NTG vs. POAG, P = 0.946). All glaucoma patients were using pressure-lowering drops; the average medication numbers were 2.1 ± 0.1 and 2.7 ± 1.5 in the NTG and POAG groups, respectively (P = 0.002).

**Table 1:** Demographics and clinical characteristics.

	Control group (n = 88)	GS group (n = 50)	NTG group (n = 73)	POAG group (n = 62)	P-value	Post-hoc P-values					
						Control vs. GS	Control vs. NTG	Control vs. POAG	GS vs. NTG	GS vs. POAG	NTG vs. POAG
Age (years)	62.7 ± 4.5	61.0 ± 12.7	59.6 ± 15.1	62.8 ± 13.0	0.080						
Sex (male, %)	38 (43.2)	23 (46.0)	33 (45.2)	30 (48.3)	0.870						
Laterality (right, %)	51 (57.9)	20 (40.0)	47 (64.3)	38 (61.3)	0.071						
BCVA (logMAR)	− 0.02 ± 0.05	0.06 ± 0.09	0.06 ± 0.15	0.07 ± 0.13	** < 0.001**	**0.001**	** < 0.001**	** < 0.001**	0.999	0.847	0.862
SE (diopter)	− 0.59 ± 1.2	− 0.77 ± 2.00	− 1.24 ± 2.61	− 1.02 ± 3.08	0.102						
IOP (mmHg)	14.9 ± 2.6	15.0 ± 2.8	12.4 ± 3.0	14.6 ± 3.3	** < 0.001**	0.998	** < 0.001**	0.870	** < 0.001**	0.841	** < 0.001**
AXL (mm)	23.7 ± 0.8	23.8 ± 0.6	24.0 ± 0.5	24.2 ± 0.9	0.560						
VF parameters											
MD (dB)	n/a	− 1.74 ± 2.49	− 6.75 ± 7.80	− 7.70 ± 7.93	**0.005**				**0.016**	**0.004**	0.737
PSD (dB)	n/a	2.54 ± 1.12	4.52 ± 4.05	7.25 ± 4.33	** < 0.001**				** < 0.001**	** < 0.001**	0.527
VFI (%)	n/a	97.4 ± 3.8	80.1 ± 24.7	78.9 ± 23.7	**0.003**				**0.006**	**0.003**	0.946

*BCVA* best-corrected visual acuity, *SE* spherical equivalent, *IOP* intraocular pressure, *AXL* axial length; *VF* visual field, *MD* mean deviation, *PSD* pattern standard deviation, *VFI* visual field index.

Significance values are in bold.

### OCT parameters

The average RNFL thickness was 96.6 ± 8.9, 95.0 ± 11.8, 77.3 ± 11.1, and 76.3 ± 15.9 μm in the control, GS, NTG, and POAG groups, respectively (P < 0.001) (Table 2). On post-hoc analysis, the RNFL of the control and GS groups was significantly thicker than that of the NTG and POAG groups; however, no significant difference was noted between the control and GS groups (P = 0.922) or between the NTG and POAG groups (P = 0.461). A comparison of sectoral RNFL thickness yielded similar results except for the nasal pRNFL thickness (P = 0.555).

**Table 2:** Optical coherence tomography parameters in each group.

	Control group	GS group	NTG group	POAG group	P-value	Post-hoc P-values					
						Control vs. GS	Control vs. NTG	Control vs. POAG	GS vs. NTG	GS vs. POAG	NTG vs. POAG
Disc parameters											
Rim area (mm^2^)	1.26 ± 0.18	1.16 ± 0.25	0.89 ± 0.21	0.79 ± 0.30	** < 0.001**	0.301	** < 0.001**	** < 0.001**	** < 0.001**	** < 0.001**	0.064
Disc area (mm^2^)	1.90 ± 0.38	2.05 ± 0.44	2.00 ± 0.49	1.85 ± 0.47	0.112						
CD ratio	0.54 ± 0.12	0.63 ± 0.13	0.71 ± 0.13	0.73 ± 0.13	** < 0.001**	**0.032**	** < 0.001**	** < 0.001**	**0.009**	**0.002**	0.900
RNFL (μm)											
Average	96.6 ± 8.9	95.0 ± 11.8	77.3 ± 11.1	76.3 ± 15.9	** < 0.001**	0.922	** < 0.001**	** < 0.001**	** < 0.001**	** < 0.001**	0.461
Superior	120.3 ± 15.8	117.1 ± 18.2	90.5 ± 22.4	87.7 ± 24.2	** < 0.001**	0.922	** < 0.001**	** < 0.001**	** < 0.001**	** < 0.001**	0.254
Temporal	70.3 ± 10.1	72.6 ± 12.4	65.9 ± 16.8	61.1 ± 18.5	**0.003**	0.927	0.425	**0.012**	0.229	**0.008**	0.274
Inferior	121.8 ± 1.3	120.4 ± 20.9	87.2 ± 23.1	86.8 ± 26.7	** < 0.001**	0.995	** < 0.001**	** < 0.001**	** < 0.001**	** < 0.001**	0.999
Nasal	70.6 ± 10.6	69.7 ± 9.5	68.5 ± 12.7	67.6 ± 11.4	0.555						

*RNFL* retinal nerve fiber layer.

Significance values are in bold.

### OCTA parameters

**Figure 2:**
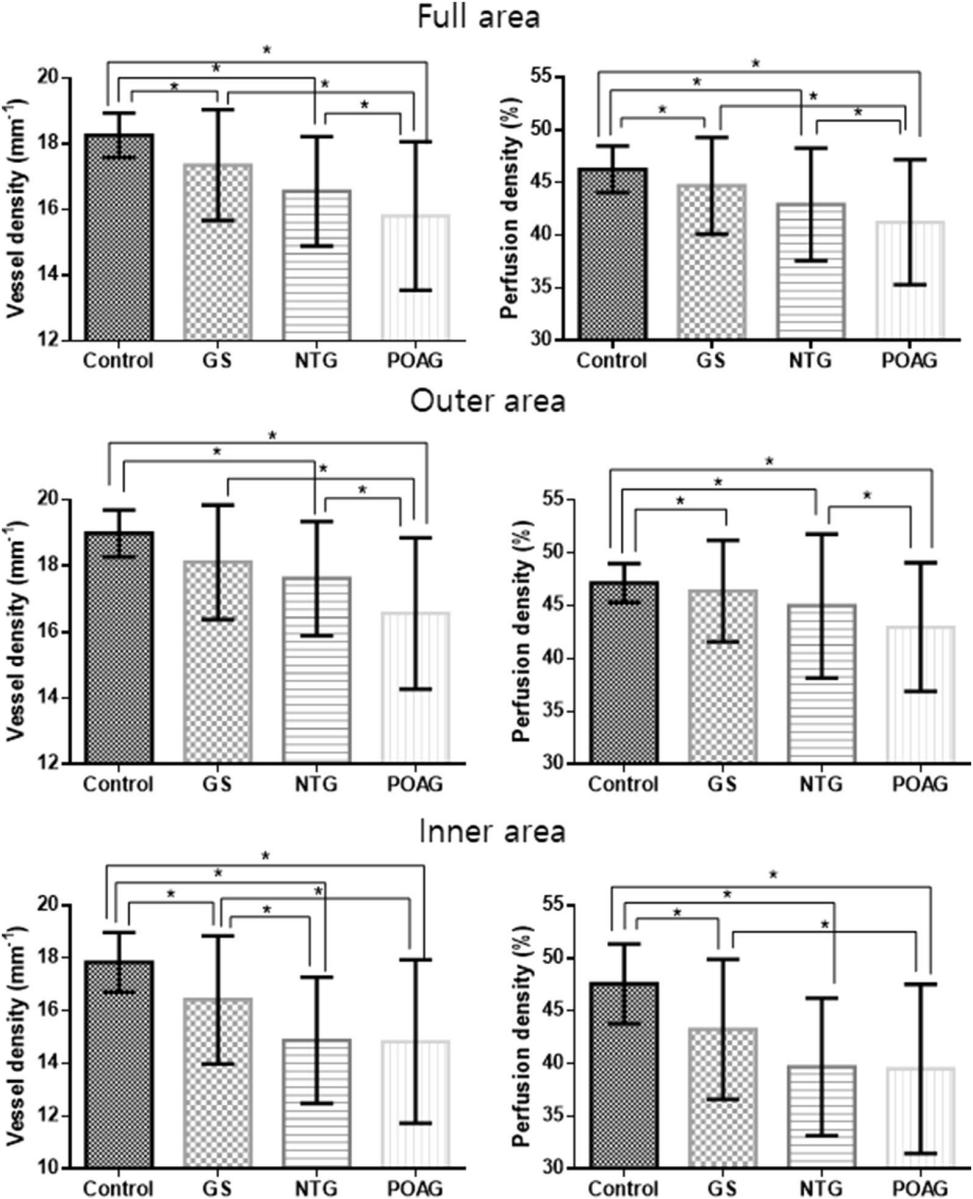
Bar graphs with a standard deviation of the peripapillary vessel density and perfusion density in each group. *Statistically significant difference.

The VDs of the full areas were 18.3 ± 0.7, 17.3 ± 1.7, 16.5 ± 1.7, and 15.8 ± 2.3 mm^−1^ in the control, GS, NTG, and POAG groups, respectively (P < 0.001) (Table 3). On post-hoc analysis, all comparisons between the two groups differed significantly except for the GS group vs. the NTG group (control vs. GS, P = 0.048; control vs. NTG/POAG, P < 0.001; GS vs. NTG, P = 0.119; GS vs. POAG, P < 0.001; NTG vs. POAG, P = 0.037) (Fig. 2). The VDs of the outer and inner areas also differed significantly among the groups (both P < 0.001); the post-hoc data were similar to those of the entire area (VD outer: control vs. GS, P = 0.074; control vs. NTG/POAG, P < 0.001; GS vs. NTG, P = 0.539; GS vs. POAG, P < 0.001; NTG vs. POAG, P = 0.001) (VD inner: control vs. GS, P = 0.030; control vs. NTG/POAG, P < 0.001; GS vs. NTG, P = 0.010; GS vs. POAG, P = 0.017; NTG vs. POAG, P = 0.999). PD comparisons yielded similar results (Table 3).

**Table 3:** Peripapillary vessel density and perfusion density in each group using optical coherence tomography angiography.

	Control group	GS group	NTG group	POAG group	P-value	Post-hoc P-values					
						Control vs. GS	Control vs. NTG	Control vs. POAG	GS vs. NTG	GS vs. POAG	NTG vs. POAG
VD											
Full	18.3 ± 0.7	17.3 ± 1.7	16.5 ± 1.7	15.8 ± 2.3	** < 0.001**	**0.048**	** < 0.001**	** < 0.001**	0.119	** < 0.001**	**0.037**
Outer	19.0 ± 0.7	18.1 ± 1.7	17.6 ± 1.7	16.6 ± 2.3	** < 0.001**	0.074	** < 0.001**	** < 0.001**	0.539	** < 0.001**	**0.001**
Inner	17.8 ± 1.1	16.4 ± 2.4	14.8 ± 2.6	14.8 ± 3.1	** < 0.001**	**0.030**	** < 0.001**	** < 0.001**	**0.010**	**0.017**	0.999
PD											
Full	46.3 ± 2.2	44.7 ± 4.6	42.9 ± 5.4	41.2 ± 5.9	** < 0.001**	** < 0.001**	** < 0.001**	** < 0.001**	0.447	**0.026**	0.256
Outer	47.2 ± 1.9	46.4 ± 4.8	45.0 ± 6.8	43.0 ± 6.1	** < 0.001**	** < 0.001**	** < 0.001**	** < 0.001**	0.724	0.072	0.228
Inner	47.6 ± 3.8	43.2 ± 6.6	39.7 ± 6.5	39.5 ± 8.0	** < 0.001**	** < 0.001**	** < 0.001**	** < 0.001**	0.102	0.091	0.999

*VD* vessel density, *PD* perfusion density.

Significant values are in bold.

### Linear regressions of OCTA and visual field parameters after adjustment for age, sex, BCVA, and axial length in the NTG and POAG groups

In the NTG group, the VDs and PDs of the full, outer, and inner areas were significantly associated with all visual field parameters, including MD, PSD, and VFI; however, the PD of the entire area was not associated with PSD (P = 0.060) (Table 4). In the POAG group, the VDs of the entire and inner areas were significantly associated with PSD and VFI but not with MD. The PDs of the entire and inner areas also showed significant associations with the VFI.

**Table 4:** Relationship between vessel density/perfusion density and visual field metrics, after adjustment for age, visual acuity, sex, and axial length in normal-tension glaucoma and primary open-angle glaucoma.

	Full area			Outer area			Inner area		
	B (95% CI)	R^2^	P-value	B (95% CI)	R^2^	P-value	B (95% CI)	R^2^	P-value
NTG									
VD									
MD	**0.08 (0.03, 0.13)**	**0.276**	**0.004**	**0.06 (0.01, 0.12)**	**0.214**	**0.029**	**0.11 (0.03, 0.18)**	**0.247**	**0.007**
PSD	− **0.17 (**− **0.26,** − **0.08)**	**0.329**	** < 0.001**	− **0.15 (**− **0.24,** − **0.05)**	**0.258**	**0.003**	− **0.21 (**− **0.34,** − **0.08)**	**0.269**	**0.003**
VFI	**0.03 (0.01, 0.04)**	**0.285**	**0.003**	**0.02 (0.01, 0.04)**	**0.220**	**0.022**	**0.03 (0.01, 0.06)**	**0.248**	**0.007**
PD									
MD	**0.30 (0.13, 0.47)**	**0.258**	**0.001**	**0.35 (0.12, 0.58)**	**0.215**	**0.003**	**0.26 (0.05, 0.47)**	**0.230**	**0.015**
PSD	− 0.31 (− 0.62, 0.01)	0.165	0.060	− **0.52 (**− **0.93,** − **0.10)**	**0.168**	**0.015**	− **0.56 (**− **0.92,** − **0.20)**	**0.262**	**0.003**
VFI	**0.10 (0.05, 0.15)**	**0.277**	** < 0.001**	**0.11 (0.04, 0.19)**	**0.207**	**0.003**	**0.09 (0.03, 0.16)**	**0.245**	**0.007**
POAG									
VD									
MD	0.07 (− 0.01, 0.15)	0.183	0.075	0.06 (− 0.02, 0.14)	0.198	0.130	0.10 (− 0.01, 0.21)	0.165	0.061
PSD	− **0.14 (**− **0.27,** − **0.01)**	**0.198**	**0.040**	− 0.11 (− 0.25, 0.02)	0.205	0.095	− **0.21 (**− **0.40,** − **0.03)**	**0.189**	**0.023**
VFI	**0.03 (0.01, 0.05)**	**0.203**	**0.032**	0.02 (− 0.01, 0.05)	0.204	0.097	**0.05 (0.01, 0.08)**	**0.216**	**0.008**
PD									
MD	0.17 (− 0.03, 0.38)	0.172	0.096	0.15 (− 0.06, 0.35)	0.194	0.160	0.26 (− 0.02, 0.55)	0.139	0.067
PSD	− 0.33 (− 0.69, 0.02)	0.182	0.064	− 0.26 (− 0.62, 0.10)	0.196	0.148	− **0.55 (**− **0.103,** − **0.07)**	**0.164**	**0.027**
VFI	**0.07 (0.01, 0.14)**	**0.194**	**0.040**	0.06 (− 0.02, 0.13)	0.200	0.124	**0.13 (0.04, 0.22)**	**0.203**	**0.006**

*NTG* normal-tension glaucoma, *VD* vessel density, *MD* mean deviation, *PSD* pattern standard deviation, *VFI* visual field index, *PD* perfusion density, *POAG* primary open-angle glaucoma.

Significant values are in bold.

## Discussion

Optic nerve damage, as a consequence of insufficient blood supply, is known to be more strongly associated with NTG than POAG, although vascular risk factors and optic nerve head perfusion may affect the development and progression of all types of open-angle glaucoma^11,12^. In contrast, mechanical nerve damage associated with high IOP would be more pronounced in POAG than in NTG. Therefore, impairment of the retinal microvasculature may result in different patterns or characteristics in the two conditions. In this study, we compared the peripapillary VDs and PDs of NTG and POAG patients with similar levels of structural and visual function damage and found that POAG patients had lower peripapillary VD and PD than NTG patients. In addition, both VD and PD were significantly associated with visual field loss.

Scripsema et al.^7^ reported that the annular perfused capillary density was 33.40 ± 6.53, 37.20 ± 3.51, and 42.45 ± 1.56% in POAG, NTG, and normal patients, respectively, which was significantly different between the groups (P < 0.01). Onishi et al.^13^ found significant decreases in the parafoveal superficial VD in POAG (40.06 ± 4.54%) and NTG (42.85 ± 5.16%) compared with healthy (48.10 ± 2.82%) eyes. We showed that the peripapillary VDs and PDs of the NTG and POAG groups were lower than those of the GS and control groups, which is consistent with previous studies. Liu et al.^14^ suggested that changes in peripapillary VD could be a reliable diagnostic parameter for detecting glaucoma with sensitivities similar to those of RNFL thickness. Additionally, peripapillary VD and PD were significantly associated with the visual field test parameters in our study, indicating that impairment of the retinal microvasculature reflects the impairment of visual function. Thus, peripapillary VD and PD measurements using OCTA may be useful for the diagnosis and follow-up of glaucoma patients.

Yarmohammadi et al.^15^ found that decreased peripapillary VD was significantly associated with the severity of visual field damage in glaucoma patients. Shin et al.^16^ reported that global and regional peripapillary VDs were significantly associated with the corresponding VF mean sensitivity. We found significant associations between peripapillary VD and PD and visual field parameters, consistent with previous studies. Concurrently, all visual field parameters were significantly associated with OCTA parameters in the NTG group with a relatively higher R^2^; however, only the PSD and VFI were associated with the VDs and PDs of the full and inner areas in the POAG group. Similarly, stronger correlations between visual function and OCTA parameters were found in the choriocapillaris in the NTG group than in the POAG group. Bhalla et al.^17^ reported that choriocapillaris flow deficit parameters were associated with visual field parameters in NTG patients, but not in POAG patients. These results suggest that vascular abnormalities are more directly associated with glaucomatous visual field damage in NTG than in POAG.

Impaired blood flow autoregulation in the optic nerve head has been speculated to be an important risk factor for glaucoma progression^18–20^. We found that the peripapillary VD and PD of the POAG group were lower than those of the NTG group when both groups exhibited similar extents of RNFL thinning and visual field impairment, although such impairment is known to be associated particularly with NTG^21,22^. Other studies have reported similar results^7,13^. This result could be partially explained by the IOP-induced deterioration of blood flow autoregulation. Chen et al.^23^ reported that the peripapillary VD of ocular hypertension patients increased after IOP reduction. Choi et al.^24^ found a decrease in VD around the optic nerve head in the vitrectomized eyes of pigs with high IOP. These results imply that an increased IOP itself also can deteriorate the autoregulation of blood flow in POAG. Under such conditions, a high IOP can also decrease ocular perfusion pressure (which is not compensated for by the autoregulation mechanism), resulting in decreased peripapillary VD and PD. This may also explain the weaker relationship between peripapillary VD and PD and visual field parameters in POAG subjects than NTG subjects in our study. According to our hypothesis, increased IOP is the main reason for glaucomatous damage in POAG, and decreased blood flow is a secondary phenomenon to the increased IOP. However, in NTG, the deterioration of blood flow autoregulation and, consequently, decreased optic nerve head blood flow might play a more direct role in glaucomatous damage. However, further studies are required to confirm this hypothesis.

Notably, the peripapillary VDs and PDs of the full and inner areas differed significantly between the control and GS groups in our study, although the RNFL thicknesses did not. Hou et al.^9^ reported that GS eyes showed significantly greater inter-eye VD asymmetry than healthy eyes, suggesting that the evaluation of such VD asymmetry may aid in screening for GS. Yarmohammadi et al.^25^ also found that GS eyes showed lower VDs in OCTA images of 4.5 × 4.5-mm fields of view centered on the optic disc than healthy eyes. These studies indicate that significant retinal microvasculature impairment was also evident in GS subjects, which is consistent with our findings. Our study also revealed that microvasculature impairment was evident without a decrease in RNFL thickness. Penteado et al.^26^ and Shoji et al.^27^ also found similar results indicating that retinal microvasculature alterations may occur before detectable inner retinal damage in POAG patients; further longitudinal studies are needed to confirm this hypothesis. Therefore, OCTA evaluation of the peripapillary microvasculature would sensitively evaluate eyes with early glaucomatous changes but no definite visual function impairment or inner retinal thinning.

This study has several limitations. First, the peripapillary VD measurements included those of the large retinal vessels, and the software did not distinguish between the microvasculature and retinal vessels. Second, we did not evaluate blood pressure or the use of glaucoma eyedrops and systemic medications, which may have affected the retinal microvasculature. Third, the heterogeneity of the GS group may have influenced our results. We included eight subjects with ocular hypertension and also possibly subjects with preperimetric glaucoma as the GS group, which could lead to a different conclusion from other studies with different GS definitions. The strength of our study is that we evaluated the peripapillary VDs of the GS, NTG, and POAG groups and compared NTG and POAG patients with similar extents of VF impairment and RNFL thinning; such reports are rare. In addition, we included only OCTA images with signal strengths exceeding 8 for accurate analyses.

In conclusion, we found that the peripapillary VDs and PDs of the GS group were lower than those of the control group, although RNFL thicknesses did not differ significantly. OCTA evaluation of microvascular changes could aid in the effective evaluation of early glaucoma patients. In addition, POAG eyes presented significantly lower peripapillary VD and PD than the NTG group, on average, despite similar extents of RNFL thinning and visual field impairment. VD and PD were significantly associated with visual field loss.

## Data Availability

The datasets used and/or analyzed during the current study available from the corresponding author on reasonable request.
